# Comparison of recall, biopsy, and cancer detection rates in the Southern Derbyshire screening programme between 2006 and 2009 using hard-copy mammography and in 2009 to 2012 following the full introduction of soft-copy reporting

**DOI:** 10.1186/bcr3293

**Published:** 2012-11-09

**Authors:** AE Turnbull, S Puri, M Bagnall, J York, S Farmer, N Horsley

**Affiliations:** 1 Breast Unit, Royal Derby Hospital, Derby, UK

## Introduction

Our programme underwent phased implementation of digital mammography, commencing soft-copy reading (SCR) in April 2009. Prior to this, all mammograms -a mixture of direct digital (DDM), computed radiography (CR) or film screen (FS) - were printed and read on multiviewers with the old films. For SCR, prior screens were available to readers but not digitised or mounted. This study was performed to evaluate recall rates, cancer detection and mammographic features assessed and biopsied, before and after SCR was introduced.

## Methods

Screening episodes between 1 April 2006 and 31 March 2012 were interrogated on NBSS. Women aged 50 to 70 years were included. Screening method, soft-copy or hard-copy reading, women recalled, cancers detected and all biopsies were analysed.

## Results

A total of 128,544 screening episodes were recorded in 6 years. The overall recall rate was identical at 2.5% between hard-copy and soft-copy reading. Seventy-two per cent of assessed women underwent biopsy in 2006 to 2009 and 79% in 2009 to 2012. The cancer detection rate was identical at 8.5/1,000 in 2006 to 2009 and 8.54/1,000 in 2009 to 2012. The number of biopsies performed was 18/1,000 women screened for FS, 18.5/1,000 for CR and 20/1,000 for DDM. No significant difference was shown in the benign/malignant ratio of calcifications biopsied in women recalled to assessment in 2006 to 2009 compared with 2009 to 2012.

## Conclusion

No significant difference was demonstrated before and after the introduction of SCR in recall rates or cancer detection. There was a nonsignificant trend towards more biopsies being performed with SCR but without additional cancers detected. Overall cancer detection rate remained high with low recall rates since adopting digital screening.

